# The pharmacokinetics of pamiparib in the presence of a strong CYP3A inhibitor (itraconazole) and strong CYP3A inducer (rifampin) in patients with solid tumors: an open-label, parallel-group phase 1 study

**DOI:** 10.1007/s00280-021-04253-x

**Published:** 2021-03-27

**Authors:** Song Mu, Chester Lin, Anna Skrzypczyk-Ostaszewicz, Iurie Bulat, Marina Maglakelidze, Viera Skarbova, Claudia Andreu-Vieyra, Srikumar Sahasranaman

**Affiliations:** 1 BeiGene USA, Inc., 2955 Campus Drive, San Mateo, CA 94403 USA; 2 Szpital LuxMed, Warszawa, Poland; 3 ARENSIA Oncology Unit, Institute of Oncology of Moldova, Chisinau, Moldova; 4 LLC ARENSIA Exploratory Medicine SRL, Research Institute of Clinical Medicine, Tbilisi, Georgia; 5 Summit Clinical Research, Bratislava, Slovakia

**Keywords:** Anticancer agents, Anticancer drugs, Clinical pharmacokinetics, CYP3A, Phase I, II and III trials, Solid tumors

## Abstract

**Purpose:**

Pamiparib is an investigational, selective, oral poly(ADP-ribose) polymerase 1/2 (PARP1/2) inhibitor that has demonstrated PARP–DNA complex trapping and CNS penetration in preclinical models, as well as preliminary anti-tumor activity in early-phase clinical studies. We investigated whether the single-dose pharmacokinetic (PK) profile of pamiparib is altered by coadministration of a strong CYP3A inducer (rifampin) or a strong CYP3A inhibitor (itraconazole) in patients with solid tumors.

**Methods:**

In this open-label, phase 1 study, adults with advanced solid tumors received either oral pamiparib 60 mg (days 1 and 10) and once-daily oral rifampin 600 mg (days 3–11) or oral pamiparib 20 mg (days 1 and 7) and once-daily oral itraconazole 200 mg (days 3–8). Primary endpoints included pamiparib maximum observed concentration (*C*_max_), and area under the plasma concentration–time curve from zero to last quantifiable concentration (AUC_0–tlast_) and infinity (AUC_0–inf_). Secondary endpoints included safety and tolerability.

**Results:**

Rifampin coadministration did not affect pamiparib *C*_max_ (geometric least-squares [GLS] mean ratio 0.94; 90% confidence interval 0.83–1.06), but reduced its AUC_0–tlast_ (0.62 [0.54–0.70]) and AUC_0–inf_ (0.57 [0.48–0.69]). Itraconazole coadministration did not affect pamiparib *C*_max_ (1.05 [0.95–1.15]), AUC_0–tlast_ (0.99 [0.91–1.09]), or AUC_0–inf_ (0.99 [0.90–1.09]). There were no serious treatment-related adverse events.

**Conclusions:**

Pamiparib plasma exposure was reduced 38–43% with rifampin coadministration but was unaffected by itraconazole coadministration. Pamiparib dose modifications are not considered necessary when coadministered with CYP3A inhibitors. Clinical safety and efficacy data will be used with these results to recommend dose modifications when pamiparib is coadministered with CYP3A inducers.

**Supplementary Information:**

The online version contains supplementary material available at 10.1007/s00280-021-04253-x.

## Introduction

Poly(ADP-ribose) polymerase 1 and 2 (PARP1/2) are enzymes involved in the regulation of nuclear processes, including DNA repair, genome stability, and programmed cell death [1]. The primary function of PARP1/2 enzymes is to detect single-strand breaks in DNA and target the breaks for repair [2]. Inhibition of PARP enzymes leads to an accumulation of unrepaired single-strand breaks, which are converted to double-strand breaks during cell division, thereby leading to genomic instability and cell death. Normal cells repair double-strand breaks in DNA using homologous recombination (HR) pathways [3]. Mutations in *BRCA1* and *BRCA2* are associated with deficiencies in HR repair [4]; thus, HR-deficient cancer cells are unable to repair double-strand DNA breaks.

Small-molecule PARP inhibitors (PARPi) have demonstrated clinical efficacy and safety for malignancies harboring *BRCA1/2* mutations such as breast, ovarian, and prostate cancer. Four PARPi (olaparib, rucaparib, niraparib, and talazoparib) are currently approved by the US Food and Drug Administration (FDA), and additional PARPi are in clinical development [5]. Though the mechanism of action for PARPi is not yet fully elucidated, PARPi have been found to directly bind and inhibit enzymatic activity of PARP, thus preventing DNA repair and trapping PARP–DNA complexes at the site of DNA damage [3].

Pamiparib (BGB-290) is an investigational, highly selective PARP1/2 inhibitor with potent PARP–DNA trapping and central nervous system penetrance in preclinical models [6, 7]. Unlike other PARPi (olaparib, rucaparib, talazoparib, and veliparib), pamiparib is not a substrate of P-glycoprotein, which can significantly restrict delivery across the blood–brain barrier [7]. A first-in-human study (NCT02361723; BGB-290-AU-002) found that pamiparib was well tolerated and showed preliminary anti-tumor activity in patients with high-grade epithelial non-mucinous ovarian cancer [8]. This study established a recommended phase 2 dose of 60 mg orally (PO) twice daily (BID) for pamiparib. Pharmacokinetic (PK) assessments from this first-in-human study demonstrated pamiparib was rapidly absorbed, with a median time to maximum concentration (*t*_max_) of 1–2 h. A dose-dependent increase in exposure was observed from 2.5 mg to 120 mg BID and from 120 mg to 160 mg once daily (QD); the geometric mean half-life of pamiparib at 60 mg BID was 13.5 h. The accumulation ratio for area under the plasma concentration–time curve (AUC) and maximum concentration (*C*_max_) at 60 mg BID were 2.37 (95% CI, 1.61–3.50) and 1.99 (95% CI, 1.47–2.69), respectively. In addition, there was a dose-dependent increase in inhibition of poly(ADP-ribose) synthesis in peripheral blood mononuclear cells from 2.5 mg to 10 mg, and the inhibition was sustained at ~ 80% for pamiparib doses of 10 mg or higher.

An in vitro phenotyping study revealed that pamiparib metabolism is primarily mediated by cytochromes P450 3A (CYP3A) and P450 2C8 (unpublished data). Many drugs are known to induce or inhibit CYP3A [9] and, consequently, could potentially alter the PK of pamiparib, thereby reducing its anti-tumor activity or increasing its toxicity [10].

This phase 1 clinical study assessed the interaction between pamiparib and the strong CYP3A inducer rifampin as well as the strong CYP3A inhibitor itraconazole. Rifampin and itraconazole were chosen because they are a preferred CYP3A inducer and inhibitor, respectively, in drug–drug interaction (DDI) studies [11, 12]. Because pamiparib has shown clastogenic activity in vitro and animal studies, consistent with its mechanism of action, this dedicated DDI study was not conducted in healthy volunteers but in patients with advanced solid tumors (NCT03994211; BGB-290-105).

## Materials and methods

### Study design and assessments

This was an open-label, parallel-group, phase 1 study consisting of a 2-part core phase and an extension phase. Only results for the core phases are reported herein (Supplementary Fig. 1). Part A of the core phase assessed the effect of the strong CYP3A inducer rifampin on the PK of pamiparib in patients with advanced solid tumors. Patients received a single dose of oral pamiparib 60 mg on days 1 and 10, and oral rifampin 600 mg QD on days 3–11. Patients fasted for at least 8 h before and at least 4 h after receiving pamiparib and rifampin. Blood samples for PK analysis were obtained on days 1 and 10 at predose, and at 0.5, 1, 2, 4, 6, 9, 12, 24, and 48 h postdose. Pamiparib plasma concentrations were determined using a validated liquid chromatography–mass spectrometry/mass spectrometry method (lower limit of quantification 1.0 ng/mL) at Covance Laboratory Services (Salt Lake City, Utah).

Part B of the core phase assessed the effect of the strong CYP3A inhibitor itraconazole on the PK of pamiparib in patients with solid tumors. All patients received a single dose of oral pamiparib 20 mg on days 1 and 7, and oral itraconazole 200 mg QD on days 3–8. The 20-mg pamiparib dose, the lowest dose strength available and one-sixth of the highest phase 1 dose (120 mg), was deemed sufficient to cover the plasma exposure increase due to complete blockade of CYP3A by itraconazole. Part B patients fasted for at least 8 h before and at least 4 h after pamiparib administration, except for day 7 upon which itraconazole administration occurred ~ 30 min after completing a meal and pamiparib administration followed within 5 min of itraconazole administration. Blood samples for PK analysis were obtained on days 1 and 7 at the same time points listed above for Part A. After completing the core phase in Part A and Part B, patients were offered participation in the extension phase, in which they were to receive pamiparib PO BID in 28-day cycles until disease progression, unacceptable toxicity, withdrawal of consent, or any other reason for discontinuation.

Adverse events (AEs) were monitored throughout the study and were graded according to the National Cancer Institute Common Terminology Criteria v5.0.

### Patient population

Patients were at least 18 years of age and had histologically or cytologically confirmed advanced or metastatic solid tumors that were refractory or resistant to standard therapy, or for which no suitable effective therapy existed. Patients had measurable disease per Response Evaluation Criteria in Solid Tumors v1.1 or Prostate Cancer Working Group-3, and an Eastern Cooperative Oncology Group performance status of 0 or 1. Adequate hematologic and organ function was required for enrollment, defined as absolute neutrophil count ≥ 1.5 × 10^9^/L, platelet count ≥ 100 × 10^9^/L, hemoglobin ≥ 9 g/dL, estimated glomerular filtration rate ≥ 50 mL/min/1.73 m^2^ by the Modification of Diet in Renal Disease study equation [13], total serum bilirubin ≤ 1.5 × upper limit of normal (ULN) or ≤ 4 × ULN if Gilbert’s syndrome or indirect bilirubin concentrations suggested an extrahepatic source of elevation, and aspartate and alanine aminotransferase ≤ 3 × ULN or ≤ 5 × ULN for patients with liver metastases. Prior treatment with a PARPi was allowed if discontinued at least 3 months prior to the first dose of pamiparib.

Key exclusion criteria included a history of hypersensitivity to rifampin or any rifamycin in Part A, a history of hypersensitivity to itraconazole in Part B, and unresolved acute effects from prior medications of grade ≥ 2 except for AEs not considered a likely safety risk (e.g., alopecia) in both parts. Use of food or drugs known to be strong or moderate CYP3A inhibitors or strong CYP3A inducers within 14 days or ≤ 5 half-lives prior to day 1 of pamiparib administration was not allowed in either Part A or Part B.

All patients provided written informed consent. The study was conducted in accordance with the principles of the Declaration of Helsinki and the International Council on Harmonisation E6 Guidelines for Good Clinical Practice. The study protocol was approved by an Independent Ethics Committee.

### Study endpoints

The primary study endpoints were *C*_max_, AUC from time zero to time of last quantifiable concentration postdose (AUC_0–tlast_), AUC from zero to infinity (AUC_0–inf_), AUC from zero to 12 h (AUC_0–12_), *t*_max_, apparent terminal elimination half-life (*t*_½_), apparent oral clearance (CL/*F*), and apparent volume of distribution (VzF). Secondary endpoints included incidence and severity of AEs; incidence of laboratory abnormalities based on hematology, clinical chemistry, and urinalysis test results; and vital sign assessment by 12-lead electrocardiogram parameters and physical examination.

### Statistical analysis

The safety population included all patients who received at least one dose of pamiparib. The PK population included all patients who received at least one dose of pamiparib and had evaluable PK data unless the patient had an AE of vomiting occurring at or before twice the median *t*_max_. Plasma PK parameters were calculated from pamiparib concentration–time profiles using standard noncompartmental methods. PK parameters were calculated using Phoenix WinNonlin v6.4 or higher (Certara, Princeton, NJ, USA). Statistical analyses were performed to assess the effect of steady-state rifampin and itraconazole on the PK of pamiparib using the combination treatment (day 10 values in Part A; day 7 values in Part B) as test and pamiparib-only treatment (day 1 values in each part) as reference (Supplementary Fig. 1). Data for each treatment part were analyzed separately. Log-transformed AUC and *C*_max_ values were analyzed using a linear mixed-effect model analysis assuming a fixed effect for treatment and a random effect for patients. The study design called for ~ 24 patients (12 in each part) to be enrolled to ensure at least 20 (10 in each part) completed the study. This sample size was based on the precedent set by other PK studies of a similar nature and was not based on power calculations.

## Results

### Patient characteristics

Between June 2019 and October 2019, 25 patients were enrolled in the study (12 patients in Part A and 13 patients in Part B). Patients were recruited from four centers in four countries. In Part A, one patient was excluded from the PK analysis population due to use of a prohibited medication; in Part B, one patient was excluded from the PK analysis population due to a grade 2 AE of vomiting, as prespecified in the statistical analysis plan. Both patients were included in the safety analysis. Patients were predominantly white females; median age in Part A was 61 years and in Part B was 68 years (Table 1). The most common tumor type was ovarian cancer (58.3% and 46.2% in Parts A and B, respectively).

**Table 1:** Summary of patient characteristics at baseline

Characteristic	Part A (*N* = 12)	Part B (*N* = 13)
Sex, *n* (%)		
Female Male	9 (75.0) 3 (25.0)	10 (76.9) 3 (23.1)
Race, *n* (%)		
White	12 (100)	13 (100)
Ethnicity, *n* (%)		
Not Hispanic or Latino	12 (100)	13 (100)
Age, years		
Mean (SD) Median Min, max	58.7 (10.7) 61.0 36, 72	62.6 (13.0) 68.0 36, 79
ECOG performance status, *n* (%)		
0 1	8 (66.7) 4 (33.3)	5 (38.5) 8 (61.5)
Tumor type, *n* (%)		
Ovarian Breast Colorectal Prostate Other	7 (58.3) 0 1 (8.3) 1 (8.3) 3 (25.0)^a^	6 (46.2) 2 (15.4) 1 (7.7) 1 (7.7) 3 (23.1)^b^

*ECOG* Eastern Cooperative Oncology Group

^a^Kidney, soft tissue sarcoma, and uterine (*n* = 1 each)

^b^Peritoneal, nasopharyngeal, and liposarcoma (*n* = 1 each)

### Pharmacokinetics

**Fig. 1:**
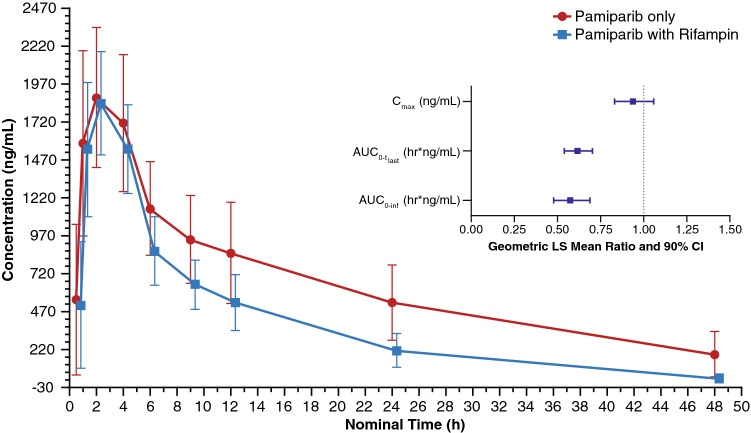
Pharmacokinetic profile of pamiparib with and without rifampin. Data presented as mean ± standard deviation. Inset: forest plot of *C*_max_ and AUC geometric least-squares mean ratios of pamiparib + rifampin: pamiparib. *AUC*_*0–inf*_ area under the plasma concentration–time curve from time zero to infinity, *AUC*_*0–tlast*_ area under the plasma concentration–time curve from time zero to last measurement, *CI* confidence interval, *C*_*max*_ maximum plasma concentration, *LS* least-squares

In Part A, coadministration of rifampin did not significantly affect pamiparib *C*_max_ or *t*_max_ (Table 2). The AUC of pamiparib, however, was reduced by rifampin, although confidence intervals overlapped at each measured time point in the concentration–time curve (Fig. 1). Following coadministration of rifampin, the geometric mean values of pamiparib AUC_0–tlast_ and AUC_0–inf_ were ~ 38 and 43% lower, respectively, than the corresponding day 1 values in this cohort (Fig. 1; inset). Administration of pamiparib in combination with rifampin also reduced the mean t_½_ of pamiparib from 12.5 h to 7.94 h (Table 2).

**Table 2:** Plasma pharmacokinetic parameters and geometric least-squares mean ratios of pamiparib with and without rifampin in the PK analysis population

Parameter	Pamiparib + rifampin (test) *N* = 11	Pamiparib (reference) *N* = 11	GM Ratio^a^ (90% CI)
*C*_max_ (ng/mL), GLSM (% CV)	1861 (20)	1986 (26)	0.94 (0.83, 1.06)
AUC_0–tlast_ (h ng/mL), GLSM (% CV)	17,762 (27)	28,841 (37)	0.62 (0.54, 0.70)
AUC_0–inf_ (h ng/mL), GLSM (% CV)	18,080 (29)	29,480 (43)^b^	0.57 (0.48, 0.69)
*t*_max_ (h), median (range)	2.0 (1.0, 4.0)	2.0 (1.0, 4.0)	NA
*t*_1/2_ (h), median (range)	7.7 (5.1, 11.4)	13.4 (6.8, 20.0)^b^	NA
CL/*F* (L/h), GM (% CV)	3.3 (29)	2.0 (43)^b^	NA

*AUC*_*0–inf*_ area under the plasma concentration–time curve from time zero to infinity, *AUC*_*0–tlast*_ area under the plasma concentration–time curve from time zero to last measurement, *CI* confidence interval, *CL/F* apparent oral clearance, *C*_*max*_ maximum plasma concentration, *CV* coefficient of variation, *GLSM* geometric least-squares mean, *GM* geometric mean, *NA* not applicable, *PK* pharmacokinetic, *SD* standard deviation, *t*_*1/2*_ time to one-half maximum plasma concentration, *t*_*max*_ time to maximum plasma concentration

^a^Ratio defined as (GLSM test/GLSM reference)

^b^*N* = 9

**Fig. 2:**
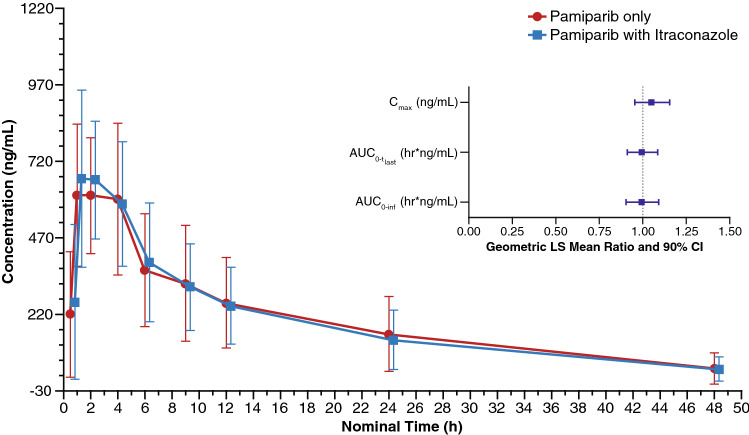
Pharmacokinetic profile of pamiparib with and without itraconazole. Data presented as mean ± standard deviation. Inset: forest plot of *C*_max_ and AUC geometric least-squares mean ratios of pamiparib + itraconazaole: pamiparib. *AUC*_*0–inf*_ area under the plasma concentration–time curve from time zero to infinity, *AUC*_*0–tlast*_ area under the plasma concentration–time curve from time zero to last measurement, *CI* confidence interval, *C*_*max*_ maximum plasma concentration, *LS* least-squares

In Part B, no significant effects on pamiparib plasma exposure (*C*_max_, AUC) were observed with coadministration of itraconazole (Table 3; Fig. 2). The geometric least-squares mean ratios for pamiparib AUC_0–inf_ and *C*_max_ (90% confidence interval [CI]) were 0.99 (0.90–1.09) and 1.05 (0.95–1.15), respectively, (Fig. 2; inset) when pamiparib was coadministered with multiple doses of itraconazole as compared to pamiparib alone. The median *t*_max_ for pamiparib was shifted to the next earlier time point, from 2 h for pamiparib alone to 1 h, when pamiparib was administered with itraconazole. Mean *t*_½_ values, however, were similar for pamiparib alone and in combination with itraconazole (11.9 and 12.0 h, respectively).

**Table 3:** Plasma pharmacokinetic parameters and geometric least-squares mean ratios of pamiparib with (test) and without (reference) itraconazole in the PK analysis population

Parameter	Pamiparib + itraconazole (test) *N *= 12	Pamiparib (reference) *N* = 12	GM ratio^a^ (90% CI)
*C*_max_ (ng/mL), GLSM (% CV)	699 (37)	665 (35)	1.05 (0.95, 1.15)
AUC_0–tlast_ (h ng/mL), GLSM (% CV)	7793 (72)	7837 (76)	0.99 (0.91, 1.09)
AUC_0–inf_ (h ng/mL), GLSM (% CV)	8381 (78)	8439 (83)	0.99 (0.90, 1.09)
*t*_max_ (h), median (range)	1.0 (0.95, 2.0)	2.0 (0.98, 4.0)	NA
*t*_1/2_ (h), median (range)	11.2 (3.7, 19.4)	9.3 (4.5, 19.1)	NA
CL/*F* (L/h), GM (% CV)	2.4 (78)	2.4 (83)	NA

*AUC*_*0–inf*_ area under the plasma concentration–time curve from time zero to infinity, *AUC*_*0-tlast*_ area under the plasma concentration–time curve from time zero to last measurement, *CI* confidence interval, *CL/F* apparent oral clearance, *C*_*max*_ maximum plasma concentration, *CV* coefficient of variation, *GLSM* geometric least-squares mean, *GM* geometric mean, *NA* not applicable, *PK* pharmacokinetic, *SD* standard deviation, *t*_*1/2*_ time to one-half maximum plasma concentration, *t*_*max*_ time to maximum plasma concentration

^a^Ratio defined as (GLSM test/GLSM reference)

### Safety

In Part A, a total of six treatment-emergent AEs (TEAEs; Table 4) were reported in five of the 12 patients enrolled (41.7%). These events consisted of anemia, palpitations, abdominal distension, catheter site inflammation, joint swelling, and upper airway secretion (*n* = 1 each). None of these were considered to be related to pamiparib, and there were no grade ≥ 3 AEs or serious AEs.

**Table 4:** Summary of adverse events in study Part A and Part B in the safety population

TEAEs	Pamiparib alone	CYP Ind/Inh alone	Pamiparib + CYP Ind/Inh	Overall
Part A. Rifampin (*N* = 12)				
All TEAEs (any grade)	1 (8.3)	2 (16.7)	2 (16.7)	5 (41.7)
Any treatment-related TEAE	0	1 (8.3)	0	1 (8.3)
Related to pamiparib only	0	0	0	0
Related to rifampin only	0	1 (8.3)	0	1 (8.3)
Related to both	0	0	0	0
Grade ≥ 3 TEAE	0	0	0	0
Serious AE	0	0	0	0
Part B. Itraconazole (*N* = 13)^a^				
All TEAEs (any grade)	2 (15.4)	2 (15.4)	2 (16.7)	6 (46.2)
Any treatment-related TEAE	1 (7.7)	1 (7.7)	1 (8.3)	3 (23.1)
Related to pamiparib only	1 (7.7)	0	1 (8.3)	2 (15.4)
Related to itraconazole only	0	0	0	0
Related to both	0	1 (7.7)	0	1 (7.7)
Grade ≥ 3 TEAE	0	1 (7.7)	0	1 (7.7)
Serious AE	0	1 (7.7)	0	1 (7.7)
Related to pamiparib only	0	0	0	0
Related to itraconazole only	0	0	0	0
Related to both	0	0	0	0

Data presented as number of patients experiencing events, *n* (%)

*AE* adverse event, *CYP* cytochrome P450, *ind* inducer, *inh* inhibitor, *TEAE* treatment-emergent adverse event

^a^In Part B, *N* = 12 for pamiparib with itraconazole

In Part B, a total of 11 TEAEs were reported in six of the 13 patients enrolled (46.2%). The most common AEs were gastrointestinal disorders; nausea and vomiting (*n* = 2 each) were the only AEs occurring in more than one patient. Among TEAEs, eructation, nausea, and erythema were considered to be related to pamiparib administered in combination with itraconazole. Vomiting, increased alanine aminotransferase, and increased aspartate aminotransferase were TEAEs considered related to pamiparib alone. One patient with stage IIIb ovarian cancer experienced a grade 4 bowel obstruction that was considered serious but not related to any study drug; however, this TEAE led to discontinuation from therapy. There were no other serious AEs and no deaths were reported in either Part A or Part B.

## Discussion

Given the prevalence of polypharmacy among patients with cancer and the prominent metabolic role of CYP3A, it is important to understand potential DDIs for pamiparib. Among other PARPi, CYP3A DDIs are a concern for olaparib [14, 15] and rucaparib [16], requiring dosage adjustments for the PARPi or even avoidance of coadministered drugs in certain cases.

To account for any potential increases in pamiparib exposure when coadministered with the strong CYP3A inhibitor itraconazole, a single dose of 20-mg pamiparib was chosen to study this interaction. Coadministration of multiple doses of itraconazole with a single pamiparib dose had no effect on the *C*_max_, AUC_0–last_, and AUC_0–inf_ of pamiparib. This observation contrasts with those reported for olaparib, for which itraconazole administration resulted in significant increases in *C*_max_ (treatment ratio 1.42) and AUC (treatment ratio 2.70), with a sevenfold AUC increase observed in one patient [14].

Coadministration of multiple doses of the CYP3A inducer rifampin with a single pamiparib dose did not affect the rate of absorption as determined by the *C*_max_ of pamiparib, but did, however, affect the extent of exposure, reducing pamiparib AUC_0–last_ and AUC_0–inf_ by 38 and 43%, respectively. Pamiparib half-life was also reduced from 13.4 to 7.7 h, due to increased clearance of pamiparib through CYP3A induction. These results are consistent with the more prominent effect from enzyme induction versus inhibition, but also suggest that CYP3A may play only a minor role in the overall metabolism of pamiparib and CYP2C8 may have a role in its metabolism as suggested by in vitro data. Rifampin is known to be a strong inducer of CYP3A and a moderate inducer of CYP2C8 [9], while itraconazole inhibition is relatively specific (~ 100-fold) for CYP3A rather than CYP2C8 [17]. For example, a previous clinical study on montelukast, a drug found to be primarily metabolized through the CYP2C8 pathway, reported no significant effects on its PK upon itraconazole coadministration, while the CYP2C8 inhibitor gemfibrozil markedly increased montelukast AUC, indicating that in vivo CYP2C8 inhibition by itraconazole was negligible [18]. If pamiparib was significantly metabolized by CYP2C8, its exposure in the presence of the CYP2C8 (and CYP3A) inducer rifampin would be expected to decrease. However, in the presence of itraconazole which does not inhibit CYP2C8 to any significant extent, only a negligible change in exposure would be noted, in line with results observed herein.

All study drugs, including single doses of 60-mg and 20-mg pamiparib administered alone or coadministered with 600-mg rifampin and 200-mg itraconazole, respectively, were well tolerated. TEAEs were infrequent, low grade, and consistent with the known safety profiles of all administered agents, including pamiparib [8, 19]. No grade ≥ 3 TEAEs or serious TEAEs related to pamiparib were reported and no treatment-related TEAEs led to study discontinuation.

In summary, coadministration of pamiparib with itraconazole, a strong CYP3A inhibitor, had no effect on pamiparib exposure. The present study, therefore, suggests that dose modifications of pamiparib may not be necessary when combined with other medications that inhibit CYP3A. On the other hand, coadministration of pamiparib with rifampin decreased the AUC of pamiparib by 38–43%, suggesting that pamiparib dosage adjustments may be necessary when strong CYP3A inducers are coadministered. With regard to the potential role of CYP2C8 in pamiparib metabolism, concomitant medications that inhibit or induce CYP2C8 are currently permitted in all pamiparib clinical trials, and their impact on pamiparib plasma exposure will be assessed using a population PK analysis approach.

The results from this DDI study will be used to perform in silico physiologically based PK (PBPK) simulations and evaluate the impact of moderate and weak CYP3A inducers, as well as CYP2C8 modulators, on the PK of pamiparib. Findings from this clinical study and the PBPK simulations, in conjunction with safety and efficacy data from clinical studies, will be used to recommend appropriate dose modifications when patients are required to take pamiparib concomitantly with inducers of CYP3A.

## Supplementary Information

Below is the link to the electronic supplementary material.

Supplementary file 1 Supp. Figure 1: Schema of core phase from open-label, parallel-group, phase 1 study. ^a^PK Samples at: Predose; 0.5, 1, 2, 4, 6, 9, 12, 24, and 48 h postdose. Abbreviations: PK, Pharmacokinetic, PO, Orally, QD, Once daily (EPS 886 KB)

## References

[1] Thomas C, Tulin AV (2013). Poly-ADP-ribose polymerase: machinery for nuclear processes. Mol Aspects Med.

[2] Dziadkowiec KN, Gasiorowska E, Nowak-Markwitz E, Jankowska A (2016). PARP inhibitors: review of mechanisms of action and BRCA1/2 mutation targeting. Prz Menopauzalny.

[3] Lord CJ, Ashworth A (2017). PARP inhibitors: synthetic lethality in the clinic. Science.

[4] Rabenau K, Hofstatter E (2016). DNA damage repair and the emerging role of poly(ADP-ribose) polymerase inhibition in cancer therapeutics. Clin Ther.

[5] Yi M, Dong B, Qin S, Chu Q, Wu K, Luo S (2019). Advances and perspectives of PARP inhibitors. Exp Hematol Oncol.

[6] Tang ZLY, Shi Z (2015). BGB-290, a novel PARP inhibitor with unique brain penetration ability, demonstrated strong synergism with temozolomide in subcutaneous and intracranial xenograft models. Cancer Res.

[7] Xiong Y, Guo Y, Liu Y, Wang H, Gong W, Liu Y, Wang X, Gao Y, Yu F, Su D, Wang F, Zhu Y, Zhao Y, Wu Y, Qin Z, Sun X, Ren B, Jiang B, Jin W, Shen Z, Tang Z, Song X, Wang L, Liu X, Zhou C, Jiang B (2020). Pamiparib is a potent and selective PARP inhibitor with unique potential for the treatment of brain tumor. Neoplasia.

[8] Lickliter JDML, Voskoboynik M (2017). Dose escalation/expansion study to investigate the safety, pharmacokinetics, food effect, and antitumor activity of BGB-290 in patients with solid tumors. Ann Oncol.

[9] US Food and Drug Administration (2020) Drug development and drug interactions: table of substrates, inhibitors and inducers. https://www.fda.gov/drugs/drug-interactions-labeling/drug-development-and-drug-interactions-table-substrates-inhibitors-and-inducers. Accessed 14 Jun 2020

[10] LeBlanc TW, McNeil MJ, Kamal AH, Currow DC, Abernethy AP (2015). Polypharmacy in patients with advanced cancer and the role of medication discontinuation. Lancet Oncol.

[11] Liu L, Bello A, Dresser MJ, Heald D, Komjathy SF, O'Mara E, Rogge M, Stoch SA, Robertson SM (2016). Best practices for the use of itraconazole as a replacement for ketoconazole in drug–drug interaction studies. J Clin Pharmacol.

[12] Srinivas NR (2016). Pharmacokinetic interaction of rifampicin with oral versus intravenous anticancer drugs: challenges, dilemmas and paradoxical effects due to multiple mechanisms. Drugs R D.

[13] Levey AS, Bosch JP, Lewis JB, Greene T, Rogers N, Roth D (1999). A more accurate method to estimate glomerular filtration rate from serum creatinine: a new prediction equation. Modification of Diet in Renal Disease Study Group. Ann Intern Med.

[14] Dirix L, Swaisland H, Verheul HM, Rottey S, Leunen K, Jerusalem G, Rolfo C, Nielsen D, Molife LR, Kristeleit R, Vos-Geelen J, Mau-Sorensen M, Soetekouw P, van Herpen C, Fielding A, So K, Bannister W, Plummer R (2016). Effect of itraconazole and rifampin on the pharmacokinetics of olaparib in patients with advanced solid tumors: results of two phase I open-label studies. Clin Ther.

[15] US Food and Drug Administration (2018) Lynparza(r) [package insert]. AstraZeneca PLC, Gaithersburg, MD; September 2018

[16] US Food and Drug Administration (2018) Rubraca(r) [package insert]. Clovis Oncology B, CO; April 2018

[17] Ong CE, Coulter S, Birkett DJ, Bhasker CR, Miners JO (2000). The xenobiotic inhibitor profile of cytochrome P4502C8. Br J Clin Pharmacol.

[18] Karonen T, Neuvonen PJ, Backman JT (2012). CYP2C8 but not CYP3A4 is important in the pharmacokinetics of montelukast. Br J Clin Pharmacol.

[19] Friedlander M, Meniawy T, Markman B, Mileshkin L, Harnett P, Millward M, Lundy J, Freimund A, Norris C, Mu S, Wu J, Paton V, Gao B (2019). Pamiparib in combination with tislelizumab in patients with advanced solid tumours: results from the dose-escalation stage of a multicentre, open-label, phase 1a/b trial. Lancet Oncol.

